# Molten Aluminum-Induced Corrosion and Wear-Resistance Properties of ZrB_2_-Based Cermets Improved by Sintering-Temperature Manipulation

**DOI:** 10.3390/ma17184451

**Published:** 2024-09-10

**Authors:** Huaqing Yi, Kezhu Ren, Hao Chen, Xiang Cheng, Xiaolong Xie, Mengtian Liang, Bingbing Yin, Yi Yang

**Affiliations:** 1 School of Materials Science and Engineering, Xiangtan University, Xiangtan 411105, China; 2 National Innovation Center for Rare Earth Functional Material, Jiangxi University of Science and Technology, Ganzhou 341000, China15970350558@163.com (X.C.); 3 Ganzhou Institute of Tungsten Industrial Technology, Ganzhou 341500, China

**Keywords:** ZrB_2_, SPS, cermet, molten aluminum, corrosion

## Abstract

During the hot dip aluminum plating process, components such as sinking rollers, pulling rollers, and guide plates will come into long-term contact with high-temperature liquid aluminum and be corroded by the aluminum liquid, greatly reducing their service life. Therefore, the development of a material with excellent corrosion resistance to molten aluminum is used to prepare parts for the dipping and plating equipment and protect the equipment from erosion, which can effectively improve the production efficiency of the factory and strengthen the quality of aluminum-plated materials, which is of great significance for the growth of corporate profits. With AlFeNiCoCr as the binder phase and ZrB_2_ as the hard phase, ZrB_2_-based ceramic composites were prepared by spark plasma sintering (SPS). SEM, EDS and XRD were used to characterize the microstructure and properties of the sintered, corroded, and abraded material samples. The density, fracture toughness, corrosion rate and wear amount of the composite material were measured. The results show that ZrB_2_-AlFeNiCoCr ceramics have compact structure and excellent mechanical properties, and the density, hardness and fracture toughness of ZrB_2_-AlFeNiCoCr increase with the increase in sintering temperature. However, when the composite material is at 1600 °C, the relative density of the sintering at 1600 °C decreases due to the overflow of the bonding phase. Therefore, when the sintering temperature is 1500 °C, the high entropy alloy has the best performance. The average corrosion rate of ZrB_2_-1500 at 700 °C liquid aluminum is 1.225 × 10^−3^ mm/h, and the wear amount in the friction and wear test is 0.104 mm^3^.

## 1. Introduction

The parts of aluminum plating equipment are eroded by contact with molten aluminum during long-term work, resulting in the formation of holes, cracks and other defects, and eventually leading to component failure which is the bottleneck problem encountered in the hot dip aluminum plating industry, Consequently, in order to guarantee safe and effective operation of the aluminum plating equipment, it would be very convenient to improve the corrosion resistance of sensitive mechanical components such as submerged rollers [1,2]. The resistance of borides to metal melt corrosion, wear and high temperature oxidation makes them suitable for use as parts in aluminized equipment [3,4,5,6]. For the ZrB_2_ ceramic material [7,8], the atomic planes form a two-dimensional network structure in the crystal structure, in which the B-B covalent bond and the Zr-B ionic bond have excellent strength, hardness and a high melting point [9,10], but the sintering temperature required for ZrB_2_ is very high, in excess of 2300 °C, so it is difficult to densify [11,12,13]. Therefore, in a ZrB_2_ single-phase material, it is difficult to meet the application requirements, and additives are needed to reduce the sintering temperature and to enable a thermal treatment to improve higher strength and structural integrity. For such a case, metal elements are usually added as a bonding phase [14,15,16,17]. The ZrB_2_-Ni material studied by Monteverde [18] uses elemental Ni as a binder, which can reduce the sintering temperature according to powder metallurgy theory, but the Ni-rich phase is easily oxidized by oxygen at high temperatures. In contrast, high-entropy alloys have the characteristics of forming a single phase with attributes of lattice, lattice distortion, slow diffusion, and mixing effect, etc., so they have excellent strength and plasticity [19,20,21,22]. Spark plasma sintering (SPS) is a new sintering technology with high thermal efficiency. Its main purpose is to put the material into the sintering mold, use the pulse current to directly generate heat energy between the powders, purify the powder surface, apply higher pressure, and prepare fine material at a lower sintering temperature [23,24,25]. Therefore, the addition of a high-entropy alloy as a bonding phase and the use of spark plasma technology for sintering [22,26,27,28] can effectively reduce the sintering temperature, increase the sintering density, and improve the situation that traditional cermet materials are easily corroded by liquid aluminum when Fe, Ni and other elements are used as binders [29,30,31]. In recent years, research on the sintering process for preparing ZrB_2_-based cermets has shown that the sintering temperature is above 1700 °C, and the AlFeNiCoCr high-entropy alloy melting point should be above 1200 °C. Therefore, exploring lower sintering temperatures under limited conditions is highly meaningful. In this paper, an AlFeNiCoCr high entropy alloy is used as the bonding phase and ZrB_2_ is used as the hard phase. The corrosion performance and service life of the zirconium diboride basic ceramic composite are improved. The preparation of the composite material is feasible. The method is simple, low cost and has good application prospects. Therefore, the design and research of this kind of boride cermet composite material is of great significance to improve the corrosion resistance of the material and solve the corrosion problem of the parts in the aluminum plating equipment.

## 2. Experimental

### 2.1. Sample Preparation

The commercial aluminum, iron, nickel, cobalt, chromium powder (purity 6499.9, 5–10 μm, Shijiazhuang Huake Metal Material Technology Co., Ltd., Shijiazhuang, China) with the same molar ratio were placed into the planetary ball mill (QXQM-1, Changsha Tencan Powder Technology Co., Ltd., Changsha, China) to prepare AlFeNiCoCr high-entropy alloy powder; the milling duration, milling rotation, and ball-to-powder were 50 h, 200 r/min, 10:1, respectively, and the milling medium was ethanol. The product was then put into the vacuum drying oven (DZ-1BCIV, Tianjin Taisite Instrument Co., Ltd., Tianjin, China) to dry, at 90 °C for 10 h. The high-entropy alloy powder and the ZrB_2_ powder (purity > 99.8%, 1–5 μm, Beijing Lichengxin Technology Co., Ltd., Beijing, China) were mixed by ball milling to prepare a mixed powder; the milling duration, milling rotation, and ball-to-powder were 5 h, 200 r/min, 5:1, respectively, and the milling medium was ethanol. It was then put into the vacuum drying oven to dry, at 90 °C for 10 h. The composition of the mixed powder is shown in Table 1.

The mixed powders were sintered to 1300 °C, 1400 °C, 1500 °C, and 1600 °C, respectively, under the vacuum state of the SPS sintering furnace at a pressure of 50 MPa. The heating rate applied was about 200 °C/min, and the holding time was 5 min. During the SPS process, the temperature was monitored by a pyrometer focused on the surface of the mold. According to the sintering temperature, four different samples were taken, named ZrB_2_-1300, ZrB_2_-1400, ZrB_2_-1500 and ZrB_2_-1600, respectively.

### 2.2. Characterization and Mechanical Tests

After polishing the samples with 400#, 800#, and 1200# sandpaper and then using a polishing machine after grinding, the scanning electron microscope (EVO MA10, Carl Zeiss AG, Oberkochen, Germany) was used, and the parameters used a 20 kV working voltage. The working principle of SEM is to use high-energy electrons to hit the surface of the material to generate secondary electrons and backscattered electrons to analyze the microstructure of the material. The OXFORD INCA type energy spectrometer (Oxford Instruments plc, Oxfordshire, UK) was used. The working principle of the energy spectrometer is to use the characteristic X-ray wavelength of the elements contained in the material to analyze the composition. The characteristic wavelength is mainly determined by the characteristic energy released when the energy level transitions. The Rigaku Ultimate IV XRD (Rigaku Corporation, Tokyo, Japan) was used. The parameters were Cu-Kα rays, 80 mA working current, 50 kV working voltage, 5°/min scanning speed, 0.02° step size, and 10~90° scanning angle. The working principle of XRD is to apply the Bragg formula: 2*dsinθ* = *nλ*, use X-rays of known wavelength to measure the *θ* angle to obtain the interplanar spacing d, for structural analysis, or use the known interplanar spacing d to measure the *θ* angle to obtain X-rays. The wavelength of the material used to obtain the elements is contained in the material.

The relative density of the sintered specimens was determined by Archimedes’ principle. The experiment uses the MH-5L Vickers hardness tester (Shanghai Hengyi Precision Instrument Co., Ltd., Shanghai, China). The Vickers hardness test method is to measure every 1 mm on the surface of the sample. A total of 5 points are measured and the average value is taken. The principle of Vickers hardness testing is to calculate the diamond traces left by the indenter of the Vickers hardness tester on the surface of the material to obtain the Vickers hardness. The parameters used were a loading load of 1 kg and a loading time of 15 s. This experiment uses the indentation method to measure the fracture toughness of the material. When measuring the hardness of a material, the indenter of the Vickers hardness tester will produce indentation and cracks when pressed down on the surface of the material. The fracture toughness of the material is obtained by the following formula [32]:

(1) $$K=0.203\times \sqrt{a}\times {\left[\frac{c}{a}\right]}^{-\frac{3}{2}}\times HV$$

In the formula, *a* is the average length of the indentation diagonal length (mm), *C* is the average length of the diagonal crack length (mm), *HV* is the Vickers hardness of the material (GPa), and a total of 5 points are taken for the test and the average value is taken.

For the corrosion tests, the specimen was cut, using a wire electric discharge machine, into pieces of 6 mm × 8 mm × 10 mm, totaling 12 pieces, (cutting three samples from each sintering temperature), the sample surfaces polished with sandpaper grade 400#, 800#, 1200# to remove surface impurity and ensure the same roughness. Finally, the samples were immersed in liquid aluminum for varied time periods. After 4, 8, and 12 days of corrosion, the samples were taken out separately, cut in half by wire-electrode cutting, 400#, 800#, 1200# sandpaper was used to polish the sample sections to remove cutting marks, and they were polished with a polishing machine after grinding to observe the corrosion of the sections. Finally, the corrosion rate was analyzed under a micrometer to accurately measure the initial thickness *a*_0_ three times at 1.5 mm intervals, and a full picture of the corroded sample was taken to accurately measure the thickness after the corrosion test under the scanning electron microscope. The corrosion rate was calculated using the following formula [33]:

(2) $$R=(a_{0}-a_{t})/2t$$

where *R* is the corrosion rate (mm/h), *a*_0_ is the initial thickness (mm), *a_t_* is the thickness after the corrosion test (mm), and *t* is the corrosion time (h).

Friction and wear experiments were carried out using a comprehensive tester (CFT-1) to evaluate the wear resistance of the materials. The experimental device was a ball disc, the friction pair was a Si_3_N_4_ ceramic ball, the friction time was 30 min, the load was 10 N, and the sliding speed was 500 r/min.

## 3. Results and Discussion

### 3.1. Microstructure

**Figure 1:**
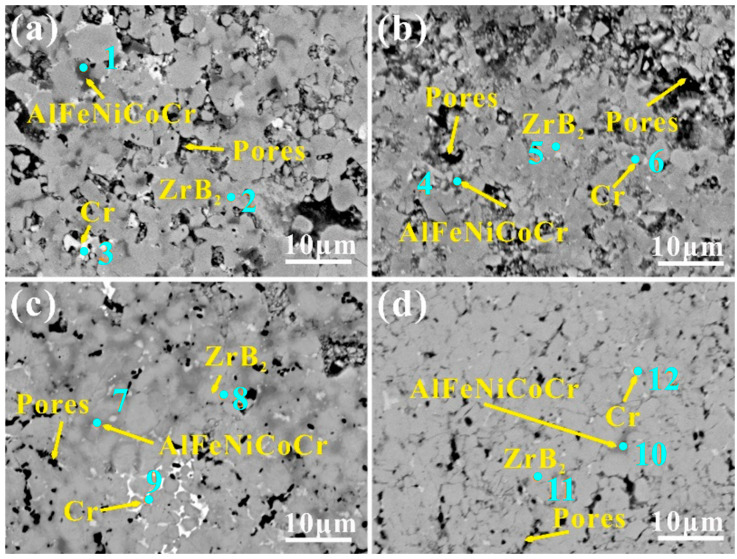
SEM micrographs of the ZrB_2_-AlFeNiCoCr cermets sintered at different temperatures. (**a**) ZrB_2_-1300, (**b**) ZrB_2_-1400, (**c**) ZrB_2_-1500, (**d**) ZrB_2_-1600. The dark gray particles are identified as AlFeNiCoCr (points 1, 4, 7, and 10), the light grey particles are ZrB_2_ (points 2, 5, 8, and 11), and the off-white particles are Cr-rich phases (points 3, 6, 9, and 12).

**Figure 2:**
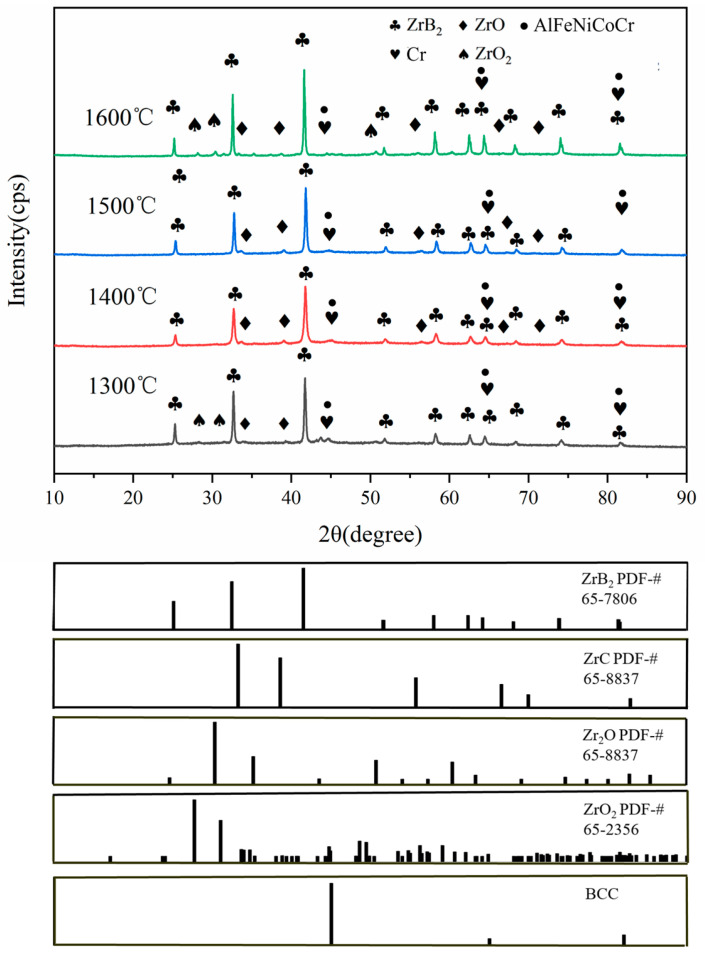
XRD patterns of ZrB_2_-AlFeNiCoCr sintered at various sintering temperatures.

The microstructures of the samples are shown in Figure 1. As the sintering temperature increases, the relative density of the sample increases significantly, while the size of the pores decreases. The increase in the sintering temperature makes the liquid phase of the high-entropy alloy filling the internal pores of the ceramic more fluid and easier to combine with the material. The relative densities of the four samples were 88.71%, 95.33%, 97.71% and 94.73%, respectively. In addition, the introduction of high-entropy alloys can cause grain boundary migration, resulting in a more uniform distribution of different particles. Figure 2 shows the XRD patterns of the ZrB_2_-AlFeNiCoCr cermets sintered at different temperatures. It was found that the peak strength and position of the ZrB_2_ (PDF-# 65-7806) phase were almost the same. The BCC (Cr-rich phase) peak of the high-entropy alloy was also medium, and the strength decreased with the increase in sintering temperature. In addition, a small amount of ZrO (PDF-# 65-8837), ZrO_2_ (PDF-# 65-2356) and ZrC (PDF-# 65-8837) are formed as ZrB_2_ reacts with oxygen impurities on the surface of the particles during the sintering process. Combining the SEM-EDS (Table 2) and XRD results (Figure 2), the dark gray particles were identified as AlFeNiCoCr (points 1, 4, 7, and 10), the light grey particles were ZrB_2_ (points 2, 5, 8, and 11), and the off-white particles were Cr-rich phase (points 3, 6, 9, and 12) in Figure 1. The high-entropy alloy was uniformly dispersed in the ceramic matrix, and it can be inferred that the sintering quality of the cermets is better.

### 3.2. Mechanical Properties

**Figure 3:**
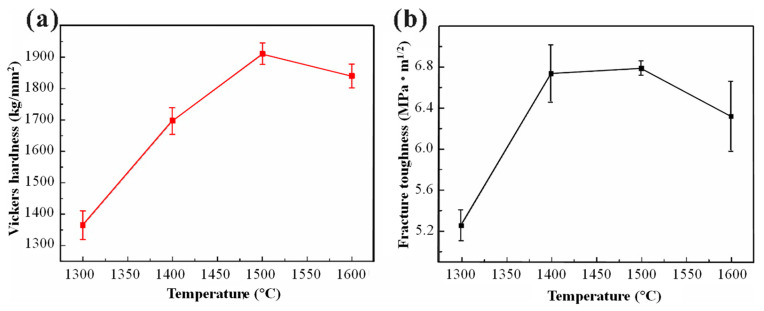
The Vickers hardness and fracture toughness results of ZrB_2_-based cermets. (**a**) Vickers hardness; (**b**) fracture toughness.

The Vickers hardness and fracture toughness results of ZrB_2_-based cermets are as shown in Figure 3. With the increase in sintering temperature, the fracture toughness of ZrB_2_-based high temperature cermets first increases and then decreases. The average fracture toughness of ZrB_2_-1500 was 6.79 MPa·m^1/2^, which is about 1.4 times that of pure ZrB_2_ ceramics (only 4.5 MPa·m^1/2^). The introduction of a high entropy alloy improves the densification and macroscopic defects in the sintering process, and further improves the fracture toughness of ZrB_2_-based cermets.

**Figure 4:**
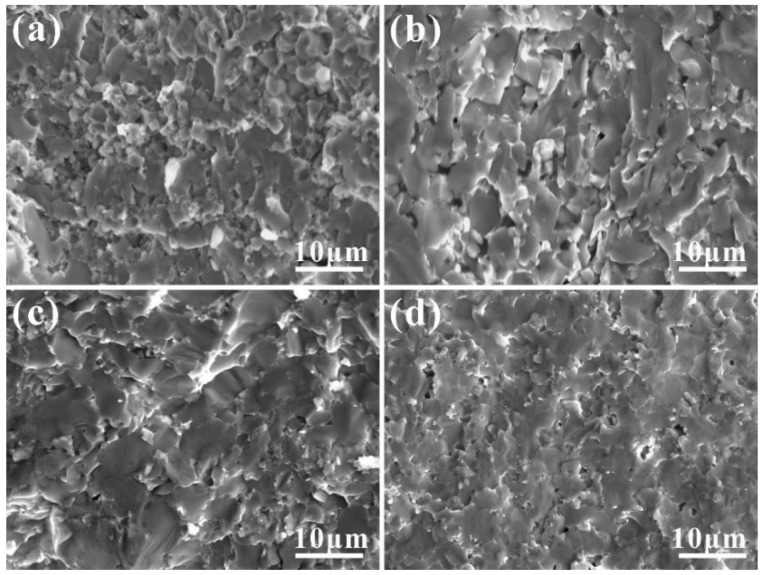
SEM micrographs of fractured surfaces of four cermets. (**a**) ZrB_2_-1300, (**b**) ZrB_2_-1400, (**c**) ZrB_2_-1500, (**d**) ZrB_2_-1600.

The SEM image of the fracture surface of the ZrB_2_-AlFeNiCoCr cermets sintered at different temperatures is shown in Figure 4. The macroscopic fracture morphology is a typical brittle fracture, with a long crystal fracture as the main fracture mode. For ZrB_2_-based cermets, the fracture of the matrix is flat and the connections between the grains are tight. The fracture morphology of the ZrB_2_-AlFeNiCoCr cermets after sintering at 1500 °C shows that the material is dense at the fracture and the ZrB_2_ particles are closely connected. The metal bonding phase is located at the boundary, fully connecting the two ZrB_2_ grains. As a result, ZrB_2_-1500 has the highest density and fracture toughness of the four materials.

### 3.3. Exposure to Molten Aluminum

**Figure 5:**
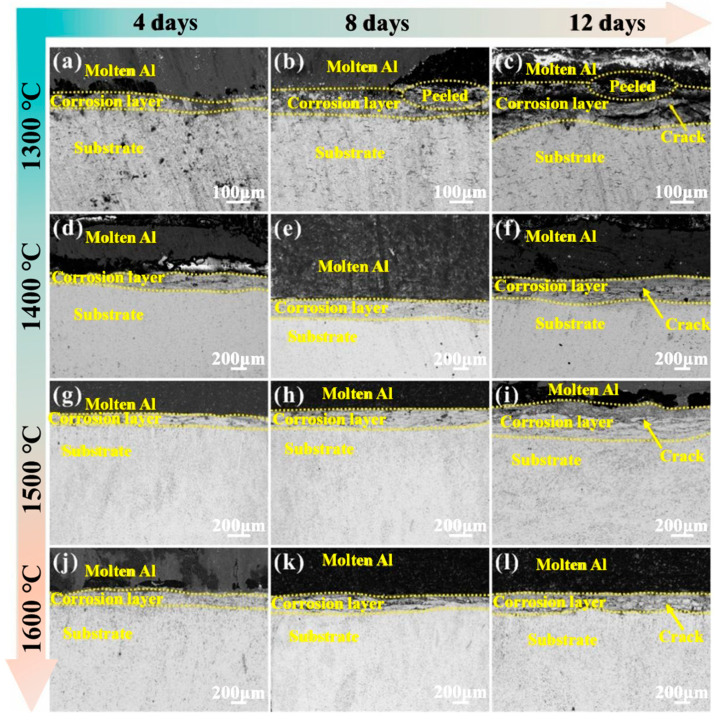
Cross-sectional morphologies of four cermets after immersion in molten aluminum at 700 °C for 4, 8, 12 days. (**a**–**c**) ZrB_2_-1300, (**d**–**f**) ZrB_2_-1400, (**g**–**i**) ZrB_2_-1500, (**j**–**l**) ZrB_2_-1600.

The microstructure in Figure 5 shows the corrosion interfaces of samples ZrB_2_-1300, ZrB_2_-1400, ZrB_2_-1500, and ZrB_2_-1600, which were exposed to molten aluminum for 4, 8 and 12 days. The cross-sectional morphologies of sample ZrB_2_-1300 show the molten aluminum had not completely wetted the base material, and there was a clear corrosion layer between the substrate and the molten aluminum phase. Molten aluminum continued to exist in the corrosion layer causing a stress concentration, and the thickness of the corrosion increased; the loose ZrB_2_ particles in the corrosion layer were abscission (Figure 5a–c). The cross-sectional morphologies of ZrB_2_-1400 show the corrosion of the material was not significant in the beginning, because molten aluminum had a large contact angle and adhesion with the composite material, which made it difficult for the molten aluminum to adhere to the surface of material, and the molten aluminum could only enter the composite material by a small number of surface pores. The thickness of the corrosion layer varied little (Figure 5d–f). The cross-sectional morphologies of sample of ZrB_2_-1500 show the density increased with the sintering temperature, and the pores reduced, resulting in the molten aluminum finding it difficult to corrode the material (Figure 5g–i). The cross-sectional morphologies of sample of ZrB_2_-1600 show that the speed at which the molten aluminum entered the interior of the material was accelerated, due to the excessive sintering temperature causing loss of the binding phase and forming more pores; the molten aluminum generally passed through the defects in the material and cracks at the stress concentration, and the pores inside the material made it easy for the molten aluminum to enter, and the corrosion layer easier to loosen (Figure 5j–l).

The molten aluminum phase and the substrate are tightly bonded at this time, indicating that the molten aluminum has been wetted by the substrate, and there is a non-dense granular reaction layer between the molten aluminum phase and the substrate. At this time, the reaction layer continues to thicken and diffuse to the substrate; the reaction layer is not dense and has many porous pores. As the etching time increases, the thickness of the reaction layer increases, and the reaction layer becomes loose. There are many cracks on the bottom plate of the photo at this time. The cracks spread from the surface of the material to the inside of the material. It can be seen that the liquid aluminum has entered the matrix along the cracks. At this time, the reaction layer closest to the aluminum layer is more porous and fluffy. The possible reason for this phenomenon is that the molten aluminum corrodes the metal bonding phase and makes the matrix material become fine particles of ZrB_2_. The reaction of liquid aluminum with the metal bonding phase produces aluminum-rich compounds, which increases the volume of the reaction layer.

**Figure 6:**
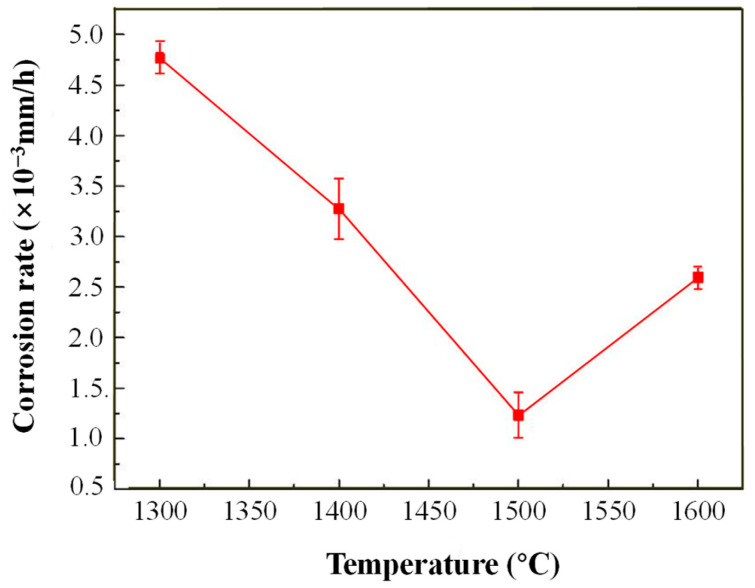
Relationship between average corrosion rate and sintering temperature after 12 days of corrosion.

Table 3 and Table 4 show the samples’ initial thickness before corrosion and samples’ thickness after the corrosion test; Figure 6 shows the corrosion rates of ZrB_2_-AlFeNiCoCr cermets in molten aluminum. The average corrosion rate of the cermets immersed in liquid aluminum for 12 days was 4.751 × 10^−3^ mm/h for ZrB_2_-1300, 3.253 × 10^−3^ mm/h for ZrB_2_-1400, 1.225 × 10^−3^ mm/h for ZrB_2_-1500, and 2.593 × 10^−3^ mm/h for ZrB_2_-1600. The ZrB_2_-1500 cermets had better corrosion resistance to molten aluminum than ZrB_2_-1300, ZrB_2_-1400, and ZrB_2_-1600 cermets. The corrosion rate of ZrB_2_-1300 cermets was relatively high, and there was no sign of acceleration or slowing down, while the corrosion rates of ZrB_2_-1400 and ZrB_2_-1600 were significantly accelerated after 12 days of corrosion. This is attributed to the loose ZrB_2_ grains on the surface, which are susceptible to the influence of molten aluminum.

### 3.4. Wear Characterization

**Figure 7:**
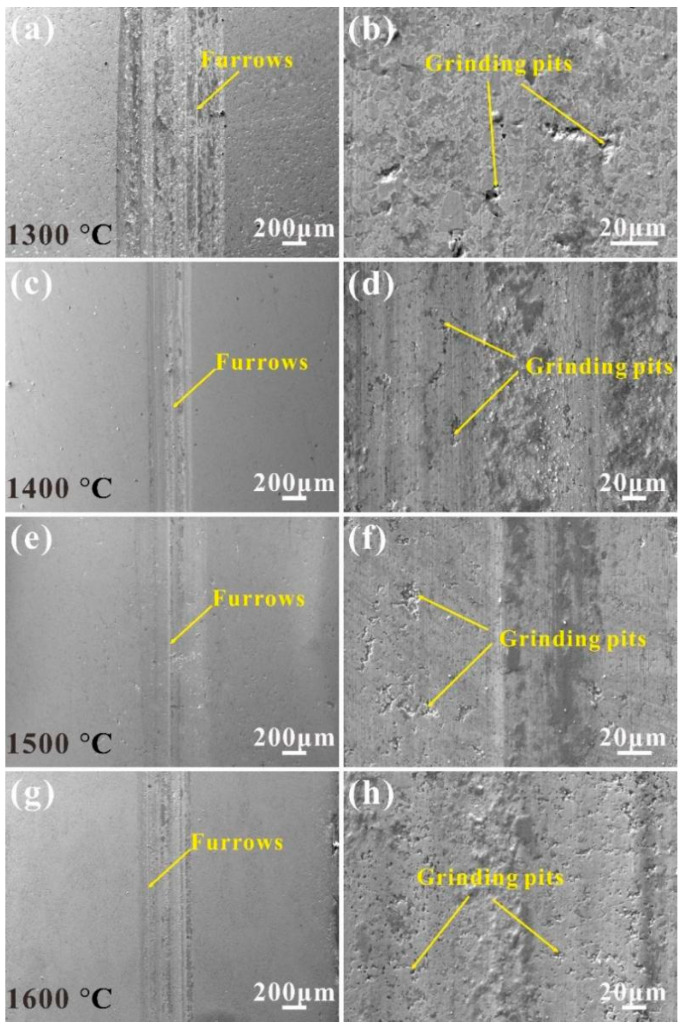
SEM micrographs showing the wear tracks of ZrB_2_-AlFeNiCoCr cermets with different temperatures: (**a**,**b**) 1300 °C, (**c**,**d**) 1400 °C, (**e**,**f**) 1500 °C, (**g**,**h**) 1600 °C.

Figure 7 shows the friction and wear morphologies of ZrB_2_-AlFeNiCoCr cermets. There was not much difference between the width and depth of the wear marks. The binder phase content of the cermets was 30%, and the density difference was small. The amount of wear on the cermets during the friction process was also similar. The average amount of wear was 0.114 mm^3^, 0.115 mm^3^, 0.104 mm^3^, 0.136 mm^3^. When the sintering temperature was 1400 °C, the bottom of the wear marks was relatively flat and accompanied by a small number of holes, which were attributed to grain shedding during the friction process. The shedding of particles promotes the formation of groove scratches during the rubbing process. At a sintering temperature of 1500 °C, the grooves in the abrasion marks were reduced, the coefficient of friction was lowered, and the wear of the material reduced. The morphology of the cermet sintered at 1600 °C was not much different from that of the cermet sintered at 1400 °C, and the same abrasive wear dominated. The increase in the sintering temperature increases the loss of the binder phase and reduces the bonding performance of the material.

**Figure 8:**
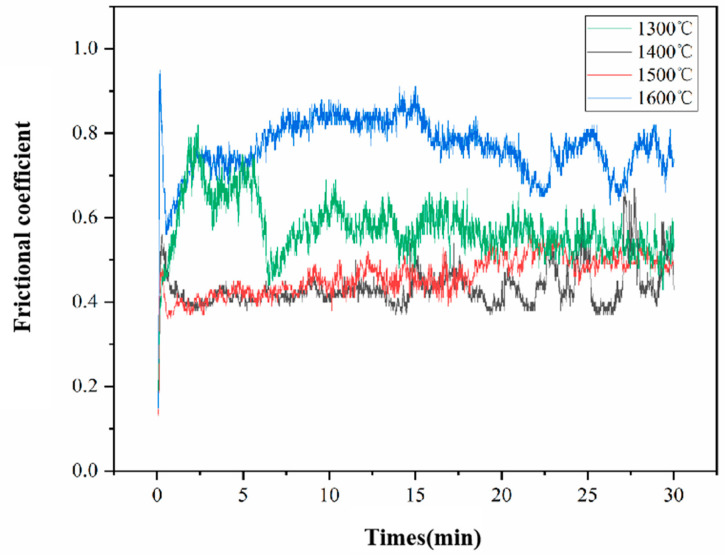
Relationship between friction coefficients and time.

The relationship between friction coefficient and friction time of the ZrB_2_-AlFeNiCoCr cermets is shown in Figure 8. The average friction coefficients were 0.57, 0.43, 0.46, 0.76, respectively. The results show that for the cermet sintered at 1300 °C, the friction coefficient sharply decreased at 5 min. This is due to the detached loose ZrB_2_ particles acting as a solid lubricant, which affects the friction. For the sample sintered at 1400 °C, the friction coefficient showed large fluctuations after 20 min and an obvious increase, indicating severe wear of the high-entropy alloy binder phase, leading to reduced wear resistance. The friction coefficient of the sample sintered at 1500 °C was relatively more stable, as the ZrB_2_ and high-entropy alloy binder phase formed strong bonding. The sample sintered at 1600 °C had the highest friction coefficient, attributed to low sintering quality, which increased the loss of the binder phase.

## 4. Conclusions

ZrB_2_-AlFeNiCoCr ceramics were prepared by spark plasma sintering at 1300 °C, 1400 °C, 1500 °C and 1600 °C. The hardness and fracture toughness were measured by the Vickers hardness indentation method, and the corrosion test of molten aluminum at 700 °C and the friction and wear test at room temperature were carried out. The experimental results show that with the increase in the sintering temperature, the hardness, density and fracture toughness of the sample first increased and then decreased, and reached the highest values at 1500 °C. The increase in sintering temperature enables the high entropy alloy binder phase to fully bond with the matrix, reducing pores and thereby increasing density. But excessively high temperatures can actually cause the bonding phase to flow out of the matrix, reducing the density. High density improved the stability of the material structure, which enhanced the hardness and fracture toughness of the materials and reduced the corrosion and detachment of the materials in molten aluminum. At 1500 °C, the hardness of ZrB_2_-based ceramics reached 1911.8 hv, the density reached 97.71%, and the fracture toughness reached 6.79 MPa·m^1/2^, which is about 1.4 times that of pure ZrB_2_-based ceramics. The corrosion rate of molten aluminum at 700 °C is 1.225 × 10^−4^ mm/h. The results show that the proper sintering temperature and the introduction of a high-entropy alloy binder phase can greatly improve the fracture toughness, hardness and density of ZrB_2_-based ceramics through microcracks and fine particles.

The high entropy alloy and boride system is complex, and different component proportions may have vastly different phase compositions and microstructures. Further optimization work should be carried out to analyze the optimal organizational and mechanical performance. Using other processing techniques, such as thermal spraying, would provide possibilities for the application of ZrB2-based cermets in other fields.

## Figures and Tables

**Table 1:** Composition parameters of ZrB_2_-AlFeNiCoCr powder (wt.%).

	ZrB_2_	Al	Fe	Ni	Co	Cr
ZrB_2_-30	70	3.21	6.63	6.99	6.99	6.18

**Table 2:** Content of different elements in different regions of Figure 2 from EDS results (at.%).

Elements	Al	Fe	Ni	Co	Cr	Zr	B
Point1	13.2	21.8	18.5	22.6	23.9	-	-
Point 2	-	1.5	0.4	0.1	0.2	46.4	51.4
Point 3	0.1	8.2	1.1	2.7	81.7	1.5	4.7
Point 4	12.8	23.3	19.5	21.3	23.1	-	-
Point 5	0.1	1.3	0.3	0.3	0.4	47.7	49.9
Point 6	0.1	8.8	1.7	2.1	82.5	1.1	3.7
Point 7	11.9	22.5	17.8	23.1	24.7	-	-
Point 8	-	1.9	0.3	0.2	0.3	48.3	49.0
Point 9	-	8.6	1.5	2.2	82.3	1.3	4.1
Point 10	12.1	22.3	19.2	22.7	23.7	-	-
Point 11	-	1.4	0.4	0.5	0.4	45.9	51.4
Point 12	0.3	9.3	1.6	3.5	79.0	2.5	3.8

**Table 3:** Samples’ initial thickness before corrosion (mm).

	1300 °C	1400 °C	1500 °C	1600 °C
Point 1	4.754	4.701	4.730	4.875
Point 2	4.831	4.641	4.787	4.704
Point 3	4.853	4.617	4.884	4.558
Average	4.813	4.686	4.800	4.746

**Table 4:** Samples’ thickness after 12 days corrosion (mm).

	1300 °C	1400 °C	1500 °C	1600 °C
Point 1	4.027	4.132	4.478	4.487
Point 2	3.970	4.273	4.606	4.316
Point 3	4.213	4.249	4.776	4.226
Average	4.129	4.218	4.620	4.386

## Data Availability

The original contributions presented in the study are included in the article, further inquiries can be directed to the corresponding authors.
